# Isolation, Identification, Prevalence, and Genetic Diversity of *Bacillus cereus* Group Bacteria From Different Foodstuffs in Tunisia

**DOI:** 10.3389/fmicb.2018.00447

**Published:** 2018-03-12

**Authors:** Maroua Gdoura-Ben Amor, Mariam Siala, Mariem Zayani, Noël Grosset, Salma Smaoui, Feriele Messadi-Akrout, Florence Baron, Sophie Jan, Michel Gautier, Radhouane Gdoura

**Affiliations:** ^1^Laboratory Research of Toxicology-Microbiology Environmental and Health, Sciences Faculty of Sfax, University of Sfax, Sfax, Tunisia; ^2^Equipe Microbiologie, Agrocampus Ouest, Institut National de la Recherche Agronomique, UMR1253 Science et Technologie du Lait et de l'œuf, Rennes, France; ^3^Department of Biology, Preparatory Institute for Engineering Studies, University of Sfax, Sfax, Tunisia; ^4^Regional Laboratory of Hygiene, Hedi Chaker University Hospital, Sfax, Tunisia; ^5^Faculty of Pharmacy, University of Monastir, Monastir, Tunisia

**Keywords:** *B. cereus* group, prevalence, phylogenetic groups, ERIC-PCR, PFGE

## Abstract

*Bacillus cereus* group is widespread in nature and foods. Several members of this group are recognized as causing food spoilage and/or health issues. This study was designed to determine the prevalence and genetic diversity of the *B. cereus* group strains isolated in Tunisia from different foods (cereals, spices, cooked food, fresh-cut vegetables, raw and cooked poultry meats, seafood, canned, pastry, and dairy products). In total, 687 different samples were collected and searched for the presence of the *B. cereus* group after selective plating on MYP agar and enumeration of each sample. The typical pink-orange uniform colonies surrounded by a zone of precipitate were assumed to belong to the *B. cereus* group. One typical colony from each sample was subcultured and preserved as cryoculture. Overall, 191 (27.8%) food samples were found positive, giving rise to a collection of 191 *B. cereus*-like isolates. The concentration of *B. cereus*-like bacteria were below 10^3^ cfu/g or ml in 77.5% of the tested samples. Higher counts (>10^4^ cfu/g or ml) were found in 6.8% of samples including fresh-cut vegetables, cooked foods, cereals, and pastry products. To verify whether *B. cereus*-like isolates belonged to the *B. cereus* group, a PCR test targeting the *sspE* gene sequence specific of the group was carried out. Therefore, 174 isolates were found to be positive. Food samples were contaminated as follows: cereals (67.6%), pastry products (46.2%), cooked food (40.8%), cooked poultry meat (32.7%), seafood products (32.3%), spices (28.8%), canned products (16.7%), raw poultry meat (9.4%), fresh-cut vegetables (5.0%), and dairy products (4.8%). The 174 *B. cereus* isolates were characterized by partial sequencing of the *panC* gene, using a Sym'Previous software tool to assign them to different phylogenetic groups. Strains were distributed as follows: 61.3, 29.5, 7.5, and 1.7% in the group III, IV, II, and V, respectively. The genetic diversity was further assessed by ERIC-PCR and PFGE typing methods. PFGE and ERIC-PCR patterns analysis allowed discriminating 143 and 99 different profiles, respectivey. These findings, associated to a relatively higher prevalence of *B. cereus* group in different foods, could be a significant etiological agent of food in Tunisia.

## Introduction

Food safety reperesents a serious public health concern. Consumption of food contaminated with pathogens and microbial by-products such as toxins could result in serious diseases. (Havelaar et al., 2012; Tewari and Abdullah, 2015; Van Cauteren et al., 2017). The contamination of foods with *Bacillus cereus* group bacteria may lead to food poisoning that usually occurs under two types of syndromes, the emetic, and/or the diarrheal syndromes (Granum, 1994). The later results from the production of several enterotoxins: Hemolysin BL (HBL), encoded by *hblA, hblB, hblC*, and *hblD*, Non-haemolytic enterotoxin (NHE) encoded by *nheA, nheB*, and *nheC*, and Cytotoxin K (CytK) encoded by *cytK*, whereas the emetic syndrome is due to a small molecular weight toxin, the cereulid, encoded by *ces*. *B. cereus* group named also *B. cereus sensu lato* includes eight closely related species: *B. anthracis, B. cereus sensu stricto, B. cytotoxicus, B. mycoides, B. pseudomycoides, B. thuringiensis, B. toyonensis*, and *B. weihenstephanensis* (Drobniewski, 1993; Guinebretière et al., 2013; Jiménez et al., 2013; Pfrunder et al., 2016). However, the most members recognized as pathogenic bacteria among *B. cereus* group were *B*. *cereus sensu stricto* an opportunistic pathogen associated with food poisoning and occasionally soft tissue infections in humans, *B. anthracis* the etiological agent of anthrax in ungulates and humans*, B. thuringiensis* an entomopathogen widely used as a biopesticide *and B. cytotoxicus*, a thermotolerant pathogen occasionally associated with food poisoning.

Furthemore, the level of sanitary risk was appeared to be dependent on the assignment of the strain to one of the seven phylogenetic groups proposed by Guinebretière et al. (2008, 2010). Based on growth temperature ranges and partial encoding pantoate-beta-alanine ligase C (*panC*) gene sequences, this affiliation allows ascribed a sanitary risk level to each phylogenetic group. Namely, the group III presents the highest risk, whereas, the lower and very lower rish was ascribed to the group V and VI, respectively. A moderate risk was associated to groups VII, IV, and II. In addition to its pathogenicity, *B. cereus sensu lato* is also known as leading to food spoilage issues (Lücking et al., 2013), due to the action of various hydrolytic enzymes even at refrigerated temperatures when psychrotolerant strains are involved (Andersson et al., 1995). *B. cereus sensu lato* are common food contaminants able to grow in a wide range of water activities, temperatures, and pH (Kramer and Gilbert, 1989). The occurrence of *B. cereus sensu lato* within the food chain may be ascribed to the use of contaminated raw materials. Moreover, technological processes such as heat treatments favor the selection of *B. cereus sensu lato* due to the endospore-forming ability of these bacteria. Furthermore, their ability to adhere on industrial surfaces and subsequently develop in the form of biofilms increase the risk of food contamination if inadequate cleaning procedures are implemented (Andersson et al., 1995). The *B. cereus* group bacteria affiliated to the psychrotrophic phylogenetic groups II and VI are also able to grow at temperatures below 7°C and can be of concern in pasteurized foods stored at chilled temperatures (Guinebretière et al., 2008; Jan et al., 2011).

Various selective solid media such as MYP (mannitol-egg yolk-phenol red-polymyxin-agar) and PEMBA (polymyxin-pyruvate-egg yolk-mannitol-bromthymol blue-agar) were used for the isolation and detection of *B. cereus* from food. The selectivity of these media is based on the hydrolysis of egg yolk lecithin and the absence of the use of mannitol by *B. cereus* besides the presence of selective compound like polymyxin (Hendriksen and Hansen, 2011).

Several molecular typing methods that rely on DNA sequence differences have been used to reveal the genetic relationship of *B. cereus* group strains (Valjevac et al., 2005; Merzougui et al., 2013a; Hill et al., 2015). Enterobacterial repetitive intergenic consensus-PCR (ERIC-PCR) was used to screen for genetic relatedness. On the other hand, the application of pulsed-field gel electrophoresis (PFGE) has been proved to be useful for the discrimination and epidemiological characterization of *B. cereus* group strains.

In Tunisia, no study has been conducted for the genotypic characterization of *B. cereus* group isolates in terms of foodborne poisoning concerns. Thus, the aim of this work was (i) to investigate the incidence and level of contamination with members of the *B. cereus* group from different kinds of foodstuffs collected in Tunisia; (ii) to assign each isolate to a phylogenetic group in order to evaluate the food safety risk level; (iii) to study the diversity of these isolates by exploring their genetic relationship through ERIC-PCR and PFGE typing methods.

## Materials and methods

### Collection of food samples

A total of 687 food samples were randomly collected from supermarkets, hotels, restaurants and private companies during the period from April 2014 to 2015. These samples represented the following products: cereal products (*n* = 103), spices (*n* = 59), pastry products (*n* = 80), cooked food (*n* = 103), canned products (*n* = 93), seafood products (*n* = 34), dairy products (*n* = 84), fresh-cut vegetables (*n* = 99), raw poultry meat (*n* = 85), and cooked poultry meat (*n* = 55). The cereals products are composed of wheat and barley flour, semolina, pasta, and couscous. The spices samples included paprika, cumin, black pepper, coriander, and curcuma. The pastry products included cakes, chocolate croissant, and ice cream. The cooked food included samples of soup, cooked pasta, omelet, minced meat sauce, grilled pepper sauce, and fries. The canned products are composed of tuna, juice, and harissa. The seafood products included fishes (sea bream and wolf fish) and clams. The dairy products are composed of milk, butter and cheese. The fresh-cut vegetables included salads composed of raw mixed vegetables and vegetables such as onion, parsley, basil, and coriander. The poultry set included samples of chicken and turkey meat, poultry legs, and wings. The cooked poultry meat samples were composed of roast and grilled chicken (meat and sausages). The different food samples were transported under complete aseptic conditions in a cool transport container to the laboratory of hygiene of Sfax (Southeast of Tunisia). The samples were analyzed within 24 h of collection.

### Culture conditions for isolation of *B. cereus* group bacteria

Ten grams of each food sample was homogenized for 1 min with 90 ml of buffered peptone water (VWR, Strasbourg, France) containing 5 g/l of lithium chloride (Prolabo, Fontenay sur bois, France) in a BagMixer stomacher (AES Laboratory, Combourg, France). Serial dilutions were prepared, and 0.1 ml of each diluted sample was streaked in MYP agar medium (Oxoid, Basingstoke, England). Plates were incubated for 24 h at 30°C. Typical colonies are pink-orange and uniform and they are surrounded by a zone of precipitation indicating lecithinase production. These colonies were presumptively identified to be *B. cereus* and enumerated with colony counter. Furthermore, from each sample, a typical colony presumed to belong to the *B. cereus* group was subcultured on BHI-YE agar (Fisher Bioblock, Illkirch, France) and incubated for 24 h at 30°C. Then, each colony was transferred into cryotubes filled with TSB broth (AES Laboratory, Combourg, France) and further incubated for 24 h at 30°C. The cultures were frozen at −80°C after addition of glycerol (Sigma Aldrich, Saint Quentin Fallavier, France), at a final concentration of 25%.

### Identification of *B. cereus* group by PCR

*B. cereus*-like isolates were grown overnight at 30°C on brain heart infusion–yeast extract (BHI-YE) (Fisher Bioblock, Illkirch, France). Genomic DNA was extracted using the Chelex extraction method as previously described (Walsh et al., 1991). Furthermore, a PCR test targeting spore structural protein gene (*sspE*) sequence specific to the *B. cereus* group (Kim et al., 2005) was carried out. This chromosomal gene sequence of a 71 bp was searched using the following primers: (5′-GAAAAAGATGAGTAAAAAACAACAA-3′) as forward degenerated primer and (5′-CATTTGTGCTTTGAATGCTAG-3′) as reverse primer (Sigma Aldrich). The amplification reactions were carried out in a PCR thermocycler (iCycler optical module 584BR, Bio-Rad, Marnes-la-Coquette, France) as follows: 4 min at 95°C, 30 cycles of 30 s at 95°C, 30 s at 59°C, and 1 min at 72°C, followed by a final extension step at 72°C for 7 min.

### *PanC* gene sequence analysis

A PCR product of a 651 bp sequence present in the *panC* gene was searched using the following primers: (5′- TYGGTTTTGTYCCAACRATGG-3′) as forward degenerated primer and (5′-CATAATCTACAGTGCCTTTCG-3′) as reverse primer. Thermal cycling was carried out in iCycler optical module 584BR (Bio-Rad) with the following run: a starting cycle of 5 min at 94°C, followed by 30 cycles of 15 s at 94°C, 30 s at 55°C, and 30 s at 72°C, and a final extension of 7 min at 72°C. Purification and sequencing of PCR products were performed by GATC Biotech AG (Biotechnology, Konstanz, Germany) using the following primer: 5′-ATAATCTACAGTGCCTTTCG-3′ (Guinebretière et al., 2008). The online algorithm (https://www.tools.symprevius.org/Bcereus/) was then used for assigning each *B. cereus* group isolate to one phylogenetic group (Guinebretière et al., 2010).

### Pulsed-field gel electrophoresis

The PFGE protocol was adapted and optimized from previous work (Merzougui et al., 2013b). Cells were grown in 5 ml BHI-YE (Fisher Bioblock) until an optical density of 0.3 at 650 nm. Bacterial suspensions were then centrifuged for at 5700 g 10 min at 4°C, and the pellet was washed in TES buffer (10 mM Tris, 1 mM EDTA, 0.5 M sucrose, pH 8) and resuspended in 400 μl TE buffer (10 mM Tris, 1 mM EDTA, pH 8) containing 10 mg/ml lysozyme (Sigma-Aldrich) before incubation at 37°C for 1 h. Four hundred microliters of lyzed cells were then added to 700 μl agarose (1% in 125 mM EDTA) and the mixture was maintained liquid by incubation at 55°C. Aliquots of 100 μl of the agarose- cell mixtures were quickly deposited into well molds in order to make blocks. The blocks were deproteinized 2 h at 55°C with 20 mg/ml proteinase K in the following buffer: 10 mM Tris, 100 mM EDTA, 10% SDS, pH 8. The proteinase K was then inactivated by washing in water and TE buffer for two to three times of 10 min respectively. To perform enzymatic digestion, blocks were dialyzed in 600 μl enzyme restriction buffer for 1 h at 4°C and then incubated in 300 μl *SmaI* solution (New England Biolabs, Hitchin, UK) (20 U in its buffer) at 25°C during 4 h. The sectioned agarose blocks were then loaded into the wells of an ultra-pure agarose gel (Gibco BRL, Paisley, Ecosse) (1.5% m/v in 0.5 X TBE buffer), and subjected to transverse alternative field electrophoresis in a CHEF-DR® II apparatus (Biorad, Hercules CA, USA), using the following parameters: 200 V, 2 s of initial switch time, 20 s of final switch time, and 18–24 h of migration at 14°C. Three deposits of lambda ladder PFGE marker (lambda DNA cI857 ind 1 Sam7, GelSyringe™, New England Biolabs, UK) were included on each **g**el. Following electrophoresis, the gel was stained with 1 μg/ml ethidium bromide, washed with deioniezd water, and visualized with an UV transilluminator (Bioblock, Illkirch, France).

### ERIC-PCR

For ERIC-PCR, the primer 5′-ATGTAAGCTCCTGGGGATTCAC-3′ (Versalovic et al., 1991) was used. The PCR was performed in a 50 μl solution containing 1 μM of primer, 5 μl of 10X PCR buffer, 0.25 mM DNTPs, 3 mM MgCl_2_, and 30 U/ml of Taq polymerase (Biolabs). The amplification reaction was performed as follows: one cycle at 95°C for 10 min followed by 4 cycles of 5 min at 94°C, 5 min at 40°C, and 5 min at 72°C and then followed by 40 cycles of 1 min at 94°C, 1 min at 55°C, and 2 min at 72°C, and the last extension at 72°C for 10 min. A 10 μl aliquot of each amplification reaction was analyzed using electrophoresis on aagarose gel (2% m/v in 1X TBE buffer) containing 1 μg/ml ethidium bromide and run in a 1X TBE buffer for 2 h at 80 V. PCR results are visualized with an UV transilluminator (Bioblock). A 1000 bp Smart DNA Ladder (Eurogentec, France) was included on the gel as marker.

### Data analysis

The PFGE and ERIC-PCR patterns were analyzed by the Bionumerics software version 6.5 (Applied Maths, Ghent, Belgium). The relationship between two given strains was scored by the Dice coefficient of similarity with an optimization of 1%, and strains were clustered by hierarchically clustering interstrain similarities based on the Unweighted Pair Group Method with Arithmetic Averages (UPMGA) clustering algorithm.

The genetic similarity between markers included on each gel was 80%. Consequently, a genetic similarity of 80% was used between isolates, which were clustered according to this similarity criterion. Therefore, we considered that two isolates presenting more than 80% similarity were the same.

## Results

### Prevalence of *B. cereus* group bacteria in food samples

A collection of 687 Tunisian food samples was screened for the prevalence of *B. cereus* group bacteria (Table 1). Overall, 191 food samples (27.8%) yielded *B. cereus*-like colonies on MYP agar, giving rise to a collection of 191 *B. cereus*-like isolates. The prevalence and contamination levels of presumptive *B. cereus* group bacteria are shown in Table 1. The bacterial concentrations were below 10^3^ cfu/g or ml in most of the food samples studied (77.5%). In 15.7% of tested samples, the *B. cereus* loads were between 10^3^ and 10^4^ cfu/g or ml, while in 6.8% of samples, the amounts exceed 10^4^ cfu/g or ml. These highest counts (above 10^4^ cfu/g or ml) were found in 20% of fresh-cut vegetables, 11.1% of cooked foods, 10.7% of cereal products and 9.7% of pastry products (Table 1). A PCR test targeting the spore structural protein gene (*sspE*) sequence, specific to the *B. cereus* group, was carried out in order to verify the belonging of each isolate to the *B. cereus* group. Therefore, 174 isolates (91.1%) were found to be positive by using this PCR test. Our molecular results showed that the recorded prevalence of *B. cereus* strains in the foodstuffs tested ranged from the highest rate of 67.7% in cereal products to the lowest of 4.8% in dairy products.

**Table 1:** Prevalence of *Bacillus cereus* group bacteria in food samples collected in Tunisia during the period from April 2014 to 2015.

**Food sample (Total no. of samples)**	**No. (%) of contaminated samples with** ***B. cereus*** **group*****-*****like bacteria within the range**			**Total no. (%) of samples comtaminated with** ***B. cereus-*****group-like bacteria**	
	**<10^3^cfu/g or ml**	**[10^3^-10^4^cfu/g or ml]**	**≥10^4^ cfu/g or ml**	**Conventional culture**	**PCR test**
Cooked food (103)	33 (73.3)	7 (15.6)	5 (11.1)	45 (43.7)	42 (40.8)
Fresh-cut vegetables (99)	4 (80.0)	0 (0.0)	1 (20.0)	5 (5.0)	5 (5.0)
Pastry products (80)	33 (80.4)	4 (9.7)	4 (9.7)	41 (51.3)	37 (46.3)
Dairy products (84)	5 (100)	0 (0.0)	0 (0.0)	5 (5.9)	4 (4.8)
Spices (59)	17 (89.5)	2 (10.5)	0 (0.0)	19 (32.2)	17 (28.9)
Canned products (54)	10 (100)	0 (0.0)	0 (0.0)	10 (18.5)	9 (16.7)
Cereal products (34)	16 (57.2)	9 (32.1)	3 (10.7)	28 (82.4)	23 (67.7)
Raw poultry meat (85)	6 (75)	2 (25)	0 (0.0)	8 (9.4)	8 (9.4)
Cooked poultry meat (55)	15 (83.3)	3 (16.7)	0 (0.0)	18 (32.7)	18 (32.7)
Seafood products (34)	9 (75)	3 (25)	0 (0.0)	12 (35.3)	11 (32.4)
Total (687)	148 (77.5)	30 (15.7)	13 (6.8)	191 (27.8)	174 (25.3)

### Genetic typing of *B. cereus* group strains isolated from food samples

We used the genetic typing of *B. cereus* strains isolated from food samples based on PCR amplification and partial sequence analysis of the *panC* gene to attribute each isolate to one of the seven genetic groups defined by Guinebretière et al. (2008). As shown in Table 2, 106 isolates (60.9%), 51 isolates (29.3%), 13 isolates (7.5%), and 4 isolates (2.3%) were assigned to the phylogenetic groups III, IV, II, and V, respectively. None of the isolates was found to be affiliated to the groups VI and VII. The group III was the most frequent phylogenetic group in our collection; accounting for 60.9% of the collection. Most isolates belonging to the group III originated from cooked samples (cooked food, pastry products, and cooked poultry products).

**Table 2:** Distribution of *B. cereus* group isolates coming from tunisian food samples according to the phylogenetic group classification defined by Guinebretière et al. (2008).

**Food sample**	**Number of *B. cereus* group isolates**	**Phylogenetic group**			
		**II**	**III**	**IV**	**V**
Cooked food	42	4	28	9	1
Fresh-cut vegetables	5	1	2	2	0
Pastry products	37	1	20	16	0
Dairy products	4	0	4	0	0
Spices	17	2	6	9	0
Canned products	9	0	8	1	0
Cereal products	23	3	11	8	1
Raw poultry meat	8	2	6	0	0
Cooked poultry meat	18	0	12	6	0
Seafood products	11	0	9	0	2
Total	174	13	106	51	4

### PFGE typing analysis

**Figure 1:**
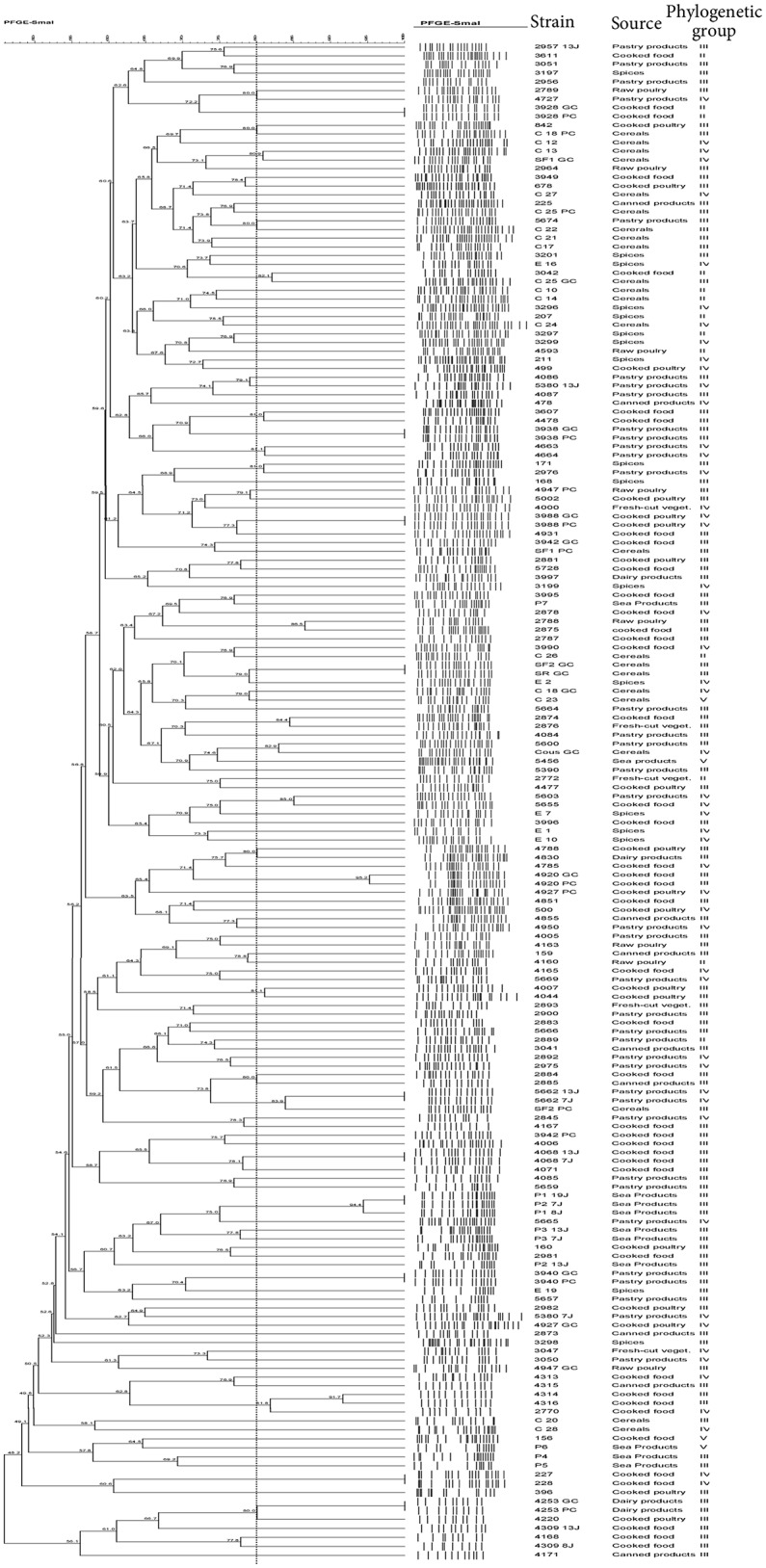
PFGE Dendogram showing the relationship between *B. cereus* isolate. The similarities between strains were evaluated using the Dice coefficient and the UPGMA clustering method. Genetic similarity between samples in duplicate is 80%.

The PFGE fingerprinting method was successful in separating the *B. cereus* group isolates coming from different foods. A remarkable polymorphism was observed inside the collection when a genetic similarity of 80% was used (Figure 1). PFGE banding patterns of *SmaI*-digested genomic DNA from 174 *B. cereus* group isolates highlighted that 58 isolates were grouped into 27 different clusters (PF1 to PF27 in Table 3), while the remaining 116 isolates were each clustered separately and considered to be genetically unrelated (Figure 1). This diversity was particularly important when the sample types varied and also within the same sample type. Among the 27 clusters obtained, 12 clusters (PF2, PF4, PF7 to PF9, PF13, PF18, PF19, PF22 to PF24, and PF26) were each associated with a specific sample type and a definite phylogenetic group. Conversely, for the 15 remaining clusters, the isolates of each cluster were not classified together based on the type of sample and/or the phylogenetic group.

**Table 3:** Relationships between ERIC-PCR and PFGE profiles within *B. cereus* isolates.

**Clusters**		**Isolates**	**ERIC-PCR profiles ^Φ^**	**PFGE profiles ^ɣ^**
ER1		5380 13J	S	D
		5380 7J	S	D
ER2	ER2(1)	5603	S	S
		5655	S	S
	ER2(2)	5662 13J	S	S
		5662 7J	S	S
ER3		4785	S	D
		5664	S	D
ER4		4927 GC	S	D
		4927 PC	S	D
ER5		4309 13J	S	D
		4309 8J	S	D
ER6		3940 PC	S	S
		3940 GC	S	S
ER7		2787	S	D
		2788	S	D
ER8		C28	S	D
		E1	S	D
ER9		E16	S	D
		P1 19J	S	S
		P1 8J	S	S
		P2 7J	S	S
		P2 13J	S	D
		E19	S	D
		E2	S	D
		E7	S	D
		E10	S	D
ER10		C22	S	D
		C25 PC	S	D
		C18PC	S	D
		C21	S	D
		C20	S	D
		C26	S	D
		C27	S	D
		C18 GC	S	D
		C14	S	D
		C17	S	D
ER11		3988 GC	S	D
		4000	S	D
ER12		4855	S	D
		4931	S	D
		3928 GC	S	S
		3928 PC	S	S
ER13		4920 GC	S	S
		4920 PC	S	S
		4788	S	S
ER14		4830	S	S
		4851	S	D
ER15		3942 PC	S	D
		3942 GC	S	D
ER16		4084	S	D
		4171	S	D
		3938 GC	S	S
		3938 PC	S	S
ER17		159	S	S
		160	S	S
		4006	S	D
		4314	S	D
		4315	S	D
		4316	S	D
ER18		4007	S	S
		4044	S	S
		3949	S	D
		3996	S	D
ER19		P3 13J	S	D
		P3 7J	S	D
		4086	S	D
ER20		2957 13J	S	D
		3201	S	D
ER21		168	S	D
		3051	S	D
ER22		5600	S	D
		5655	S	D
		5728	S	D
		3997	S	D
ER23		2875	S	D
		3041	S	D
		2874	S	D
ER24		4167	S	D
		4477	S	D
ER25		2873	S	D
		2876	S	D
ER26		4253 GC	S	S
		4253 PC	S	S
		4220	S	S
ER27		4068 13J	S	S
		4068 7J	S	S
		2981	S	D
ER28		4160	S	D
		4593	S	D
ER29		2900	S	D
		2956	S	D
ER30		2892	S	D
		3047	S	D
		3296	S	D
ER31		3199	S	D
		3988 PC	S	D
ER32		2772	S	D
		3297	S	D
ER33		499	S	D
		5456	S	D
ER34		SF2 GC	S	S
		SR GC	S	S
ER35		2975	S	D
		2976	S	D
ER36		227	S	S
		228	S	S
		478	S	D
ER37		P7	S	D
		SF1 PC	S	D
ER38		2883	S	D
		2884	S	D
PF1		2789	D	S
		4727	D	S
PF2		3928 GC	S	S
		3928 PC	S	S
PF3		842	D	S
		C18 PC	D	S
PF4		C13	D	S
		SF1 GC	D	S
PF5		5674	D	S
		C22	D	S
PF6		3042	D	S
		C25 GC	D	S
PF7		3607	D	S
		4478	D	S
PF8		3938 GC	S	S
		3938 PC	S	S
PF9		4663	D	S
		4664	D	S
PF10		171	D	S
		2976	D	S
PF11		3988 GC	D	S
		3988 PC	D	S
PF12		2788	D	S
		2875	D	S
PF 13		SF2 GC	S	S
		SRGC	S	S
PF14		2874	D	S
		2876	D	S
PF15		5600	D	S
		COUS GC	D	S
PF16		5603	S	S
		5655	S	S
PF17		4788	S	S
		4830	S	S
PF18		4920 GC	S	S
		4920 PC	S	S
PF19		4007	S	S
		4044	S	S
PF20		2884	D	S
		2885	D	S
PF21		5662 13J	S	S
		5662 7J	S	S
		SF2 PC	D	S
PF22		4068 13J	S	S
		4068 7J	S	S
PF23		P1 19J	S	S
		P2 7J	S	S
		P1 8J	S	S
PF24		3940 GC	S	S
		3940 PC	S	S
PF25		4314	S	S
		4316	S	S
		2770	D	S
PF26		227	S	S
		228	S	S
PF27		4253 PC	S	S
		4253 GC	S	S
		4220	S	S

ɣ *Based on the PFGE Dendogram shown in Figure 1*.

**Figure 2:**
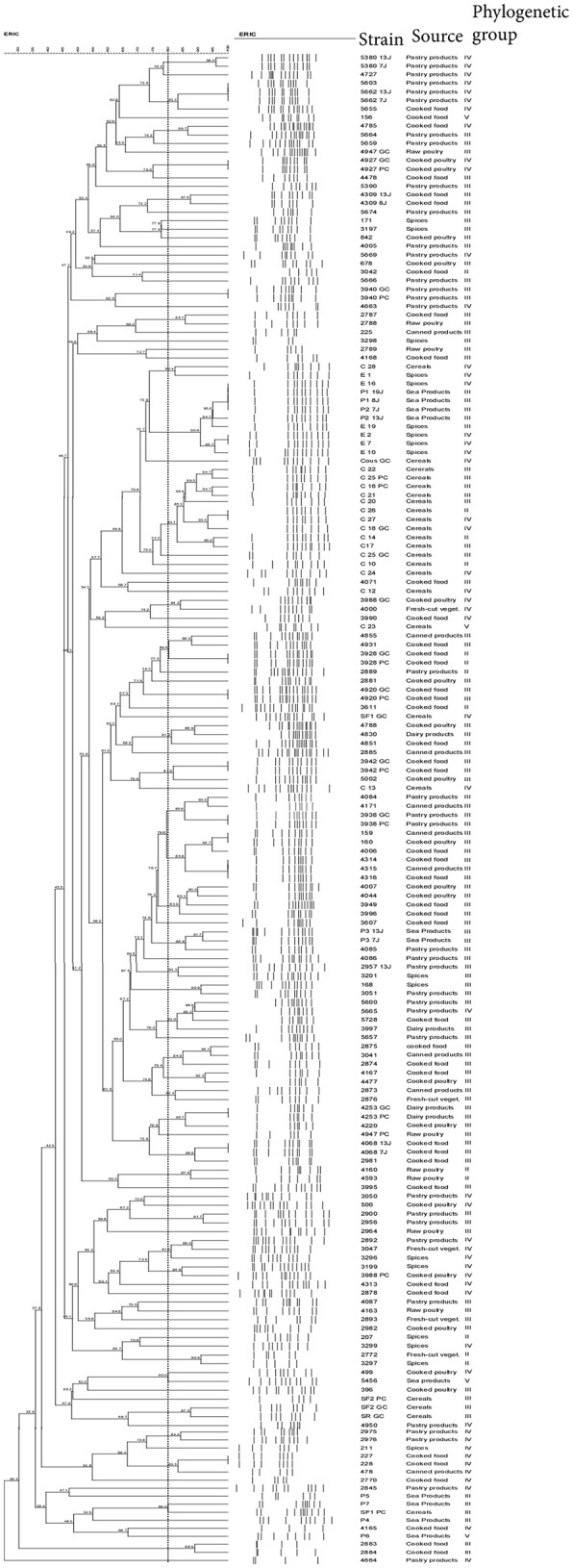
ERIC-PCR Dendogram showing the relationship between *B. cereus* isolate. The similarities between strains were evaluated using the Dice coefficient and the UPGMA clustering method. Genetic similarity between samples in duplicate is 80%.

Φ *Based on the ERIC-PCR Dendogram shown in Figure 2*.

*ER, ERIC-PCR cluster; PF, PFGE cluster; S, Similar; D, Different*.

*Depending to their PFGE profiles, The four isolates belonging to ER2 cluster are divided into two sub-clusters: ER2 (1) and ER2 (2). Each sub- cluster displayed identical PFGE profiles but different from the other one*.

### ERIC-PCR typing analysis

Using 80% similarity criteria, 61 isolates were unclustered while the remaining 113 isolates were grouped into 38 clusters (Figure 2). In this case, the isolates distribution in clusters was done independently of the **sampling** origin and the phylogenetic group of strains. The genetic relationships of isolates and different clusters obtained from the ERIC-PCR and PFGE typing methods are summarized in Table 3. The present study reveals that *B. cereus* isolates belonging to clusters (ER6-PF24; ER13-PF18; ER26-PF27 and ER34- PF13) showed concordant typing results in ERIC-PCR and in PFGE. A diverging typing results were obtained for the ramaining isolates. As shown in Table 3 the majority of clusters with identical ERIC profiles displayed at least one strain exhibited a different PFGE profile [ER2(1)-PF16; ER12-PF2; ER14-PF17; ER16-PF8; ER18-PF19; ER27-PF22; ER9-PF 23; ER36-PF26; ER1; ER7; ER8; ER10; ER11; ER15; ER35; ER37; ER38; ER3 to ER5, ER19 to ER25, and ER28 to ER33). Conversely, some strains with different ERIC profiles presented identical PFGE profile (PF1, PF3 to PF7, PF9 to PF12, PF14 to PF15, and PF20).

## Discussion

*B. cereus sensu lato*, a widespread pathogen, can be found in different types of foodstuffs. According to the (Public Health England, 2009), the allowable level of *B. cereus* group bacteria in food is below 10^3^ cfu/g or ml. However, doses as low as 10^3^ *B. cereus* cfu/g or m1 of food sample may be sufficient to cause food poisoning (Gilbert and Kramer, 1986; Stenfors Arnesen et al., 2008). In fact, it is important to note that food products may be easily contamined due to handling and storage conditions or to inefficient cleaning and sanition of the equipment. Thus, the bacterial load may rapidly rise and reach the dangerous level (up to 10^4^ cfu/g or ml). This later differs depending on the standard guidelines: the amount of *B. cereus* is considered unsafe for consumption with only >10^5^ cfu/g or ml in UK's guidelines(Public Health England, 2009) and ≥ 10^4^ cfu/g or ml in Food Standards Australia New Zealand (Food Standards Australia New Zealand, 2001). Our study shows that *B. cereus*-like bacteria were isolated from many different food samples. In 77.5% of the tested samples, the loads were below 10^3^ cfu/g or ml (the UK's permitted level, Public Health England, 2009), wheras, in only 6.8% of the samples, the counts were above 10^4^ cfu/g or ml (the Australia New Zealand's hazardous level, Food Standards Australia New Zealand, 2001). The high counts found in fresh- cut vegetables, cooked food, and pastry products were similar to those reported by Rosenquist et al. (2005) in fresh cucumbers and tomatoes, heat-treated products, cake custard, and desserts. The high contamination level of processed foods may result from contamination of raw materials and the subsequent resistance of spores to thermal or other manufacturing processes. Slow cooling and extended storage at room temperature allow the spores to germinate and re-grow (Borch and Arinder, 2002; Ankolekar et al., 2009). Biofilms of *B. cereus* group bacteria present on the surface of the pipelines and other processing materials such as storage tanks can be a source of contamination of the food being processed (Faille et al., 2014).

The amounts of *B. cereus* group bacteria in cereal products are in accordance with the contamination level reported by Lee et al. (2009) which ranged from 30 to more than 2.4 10^3^ MPN/g. The presence of *B. cereus* group bacteria in cereal products could be ascribed to the abilities of these microorganisms to survive under high temperature and dried conditions (Claus and Berkeley, 1986) as well as to the packaging process of this type of food (Väisänen et al., 1991). The contamination of seafood products was similar to that was reported by Rahmati and Labbe (2008), i.e., a contamination level ranging from 36 cfu/g to 1.1 10^3^ cfu/g. All canned and dairy product samples contaminated with *B. cereus*-like bacteria were considered as satisfactory (counts below 10^3^ cfu/g or ml). However, a study conducted in India reported much higher *B. cereus* counts, i.e., above 10^5^ cfu/g in 10% of milk product samples (Bedi et al., 2005). Our data show that the contamination level were below 10^3^ cfu/g for most spice samples analyzed (89.5%). Only two samples (10.5%) had counts ranging from 10^3^ cfu/g to 10^4^ cfu/g. Similar counts have been reported in a Turkish study on spices (Aksu et al., 2000). However, Kneifel and Berger (1994) detected that some spices had *B. cereus* counts as high as 10^5^ cfu/g. Therefore, the use of such contaminated spices as additive in food may represent a risk in the case of inadequate heat treatment. The counts of *B. cereus* group bacteria in 75 and 83.3% of raw poultry meat and cooked poultry meat samples, respectively, were below 10^3^ cfu/g. None of these samples showed bacterial amounts above 10^4^ cfu/g. These bacterial loads were slightly lower than those found in the Sudershan et al. (2012) study focusing on the microbiological hazards in poultry based street food. They published a contamination level ranging from 2.4 to 2.6 10^3^ cfu/g. The amounts of *B. cereus* group bacteria in such food may be related to contaminations vehiculated by food additives added to poultry meat during cooking (Floristean et al., 2007) or to cross-contaminations by the food handlers, the cooking utensils or the environment. The presence of *B. cereus* group bacteria in raw chicken meat may be due to the contamination during slaughtering, processing delivery, transportation, or storage of the meat. Inadequate temperatures of cooking or storage of the raw poultry may also facilitate bacterial growth (Floristean et al., 2007).

As shown in Table 1, PCR amplification of the *sspE* gene revealed that the presumptive *B. cereus* group strains isolated from fresh-cut vegetables, raw, and cooked poultry meat samples were all confirmed to belong to the *B. cereus* group. For all the other contaminated samples, at least one of each type of foodstuffs was found negative by PCR. Therefore, these results indicate that the conventional culture method is not sufficient to detect *B. cereus* group bacteria. The high bacterial prevalence in the tested cereal samples (67.7%) is comparable to the finding obtained by Lee et al. (2009), showing that 76% of the cereals they analyzed was contaminated with *B. cereus* group bacteria. The prevalence of *B. cereus* group bacteria in dairy products and in fresh-cut vegetables (4, 8 and 5%, respectively) was lower than that recorded in other studies (Samapundo et al., 2011; Merzougui et al., 2013b). Therefore, a higher incidence, ranging fom 30 to 40%, was observed in fresh-cut vegetables (Samapundo et al., 2011) and up to 40% in dairy products (Merzougui et al., 2013b). The variability of bacterial prevalence in dairy products could be attributed to the manufacturer practices and to the storage conditions of the products (Tewari and Abdullah, 2015). The incidence of the *B. cereus* group in raw and cooked poultry meat was 9.4 and 32.7%, respectively. This finding is in agreement with the studies of Floristean et al. (2007) and of Aklilu et al. (2016) showing a higher prevalence of the bacteria in cooked poultry compared to raw poultry samples. The incidence levels reported in the literature are quite variable for spices and for seafood products. Compared to our data, a higher incidence was reported in the spices analyzed by Merzougui et al. (2013b) and Kneifel and Berger (1994) with 32.8 and 49.37%, respectively. Conversely, Shah et al. (1996) reported a lower *B*. *cereus* group incidence, with 20% contaminated spice samples in India. For seafood products, the incidence was almost the same than that recorded by Das et al. (2009) (36.7%), whereas Rahmati and Labbe (2008) reported a lower prevalence in seafood products (17.9%). *B. cereus* group bacteria have also been identified in 46.3% of the pastry products, 40.8% of the cooked food samples, and 16.7% of the canned products. Our results are in agreement with other studies where the *B. cereus* group was found in heat-treated food such as sauces, soups, pasta, cooked rice (Rosenquist et al., 2005), and pizza (Eglezos et al., 2010), in pastry products and desserts (Rosenquist et al., 2005; Wijnands et al., 2006), and in canned products (Oranusi et al., 2012).

In order to assign *B. cereus* group isolates to different phylogenetic groups (I to VII), partial *panC* gene sequence of each isolates was compared with those of reference strains using a Sym'Previous software tool. Groups II and VI are psychrotrophic, groups III, IV, and VII are mesophilic and group V has an intermediate behavior (Guinebretière et al., 2008). The highest risk of foodborne illnesses was associated to the group III, a moderate risk to the groups VII, IV, and II, a lower risk to the group V and a very low risk to the group VI (Guinebretière et al., 2008). None of the strains within the group I was isolated from foods (Guinebretière et al., 2008**)**. In this study, the phylogenetic group III was the most frequent in our collection (60.9%). The high prevalence of isolates belonging to group III might be associated with food processing and/or storage of cooked food, often at abusive temperatures as well as prolonged storage at a high temperature (Six et al., 2012). Therefore, these conditions are assumed to select strains of group III which spores are characterized by a high heat resistance and an ablity to germinate and grow at 15–45°C (Carlin et al., 2010). This group is one of the most cytotoxic groups that could probably present a potential risk for consumers (Guinebretière et al., 2010). Overall, 29.3% isolates were assigned to the group IV that represents a high risk of food poisoning (Guinebretière et al., 2010). Compared to the group III, a lower occurrence of group IV isolates was highlighted. This could be explained by the fact that strains belonging to the group IV are more susceptible to heat treatments than those belonging to the group III (Afchain et al., 2008; Carlin et al., 2010). The remaining isolates (7.5%) were affiliated to the psychotrophic phylogenetic group II while any strain was classified into the phylogenetic group VI, presumably due to the higher heat-sensitivity of the spores of psychotrophic bacteria and considering that the heat treatments applied were severe. Isolates belonging to the group V, which are described as having an intermediate temperature growth range, were rare in our collection, presumably because of their low prevalence in raw materials and ingredients (Afchain et al., 2008). As shown in table 2, each food samples type could be contaminated by a mixture of various genetic groups. Therefore, each step in a processing chain, comprising cooking, blending, and addition of ingredients, may represent a secondary way of contamination of the food product (Afchain et al., 2008). Storage at chilled temperatures may represent an additional risk since psychrotrophic *B. cereus* group strains which have resisted heat treatments are able to grow at low temperature (Afchain et al., 2008; Guinebretière et al., 2008).

This is the first study in Tunisia with the specific aim to identify and determine the occurence of *B. cereus* group bacteria in food. The genotypic typing of *B. cereus* group isolated in Tunisia carried out by ERIC-PCR and PFGE as well as their assignment to phylogenetic groups allowed a better assessment of the biodiversity of the food isolates. The analysis of the PFGE and ERIC-PCR patterns allowed discriminating 143 and 99 profiles, respectively, from a total of 174 strains tested. Our finding emphasizes the efficiency of PFGE and ERIC-PCR for epidemiological studies. In this study, the use of PFGE and ERIC-PCR for typing *B. cereus* group strains highlighted that some genetic relatedness are not correlated with sample origins and/or phylogenetic group. This diversity could suggest various origins of contamination such as raw materials, equipments and processing (Andersson et al., 1995; Altayar and Sutherland, 2006). As shown in Table 3, the majority of clusters with identical ERIC-PCR profiles displayed at least one strain exhibiting a different PFGE profile. Our finding is in agreement with previous studies, including a study on *Salmonella* (Fendri et al., 2013) and another on *B. cereus* group (Merzougui et al., 2013a), which showed that PFGE presents a higher discriminatory power than ERIC-PCR. However, ERIC-PCR appeared to be more discriminative than PFGE when strains with different ERIC-PCR profiles showed identical PFGE profiles. Therefore, the level of discrimination is different for each method. Consequently, PFGE and ERIC-PCR each provide its specific measure of genetic diversity (Kidd et al., 2011). In principle, ERIC-PCR reveals a profile of DNA fragments of different sizes depending on the genomic locations of specific repetitive sequences, while PFGE reveals restriction site polymorphisms arising from the genomic locations of *SmaI* sites. Thus, ERIC-PCR may have a complementary role to PFGE typing for a better assessment of the biodiversity of *B. cereus* group strains in food products.

Several studies emphasize that MLST most likely allows detecting more variations in the bacterial genome than PFGE and is therefore more discriminatory (Revazishvili et al., 2004; Harbottle et al., 2006; Ehling-Schulz and Messelhäusser, 2013). However, the seven genes, which MLST is based on, do not mutate fast enough to show a genetic diversity signature (Ågren et al., 2017). Indeed, it is possible that critical strain-differentiating nucleotide substitutions, insertion or deletion were localized outside the seven loci analyzed by MLST (Noller et al., 2003). Therefore, PFGE and ERIC-PCR has the advantage of randomly “probing” the entire genome, whereas MLST only analyzes nucleotides within targeted genes. Another major drawback of MLST-based approaches is the requirement of substantial hands-on-time for the sequencing of seven genes per strain and subsequent data analysis, which limits its applicability for high throughput studies (Ehling-Schulz and Messelhäusser, 2013). Finally, the comparison of these major three typing methods is warranted for each bacterial species before one makes a decision concerning which method has the superior discriminatory ability. As perspective, the use of MLST would be of considerable interest for a better evaluation of the genetic diversity inside the present bacterial collection.

Although the loads in most of the tested samples are at levels considered to be safe for consumption, the relatively higher prevalence as well as the diversity observed among *B. cereus* group isolates contaminating Tunisian food could represent a significant health risk in Tunisia. Furthermore, the phylogenetic group III, which is recognized as being generally highly cytotoxic was the most frequent in our collection, and especially in cooked samples (cooked food, pastry products, and cooked poultry products). Therefore, serious attention should be devoted to the sanitary and temperature conditions under which foods are prepared, cooked, and marketed in Tunisia. An overall assessment of the sanitary risk potential linked to *B. cereus* group bacteria needs additional research, including cytotoxicity tests. These future works will allow confirming the need of including the *B. cereus* group in disease control and food safety programs, as well as in routine clinical and food quality control laboratories in Tunisia.

## Author contributions

MG-B: conception and design of the work and writing of the manuscript; MS and MZ: interpretation of the data, writing, and review of the manuscript; NG: acquisition, analysis, and interpretation of data; SS and FM-A: supervision of laboratory work and cultures; FB and SJ: contribution to the writing and review; MG and RG: supervision of the project, important intellectual contributions and final approval of the version to be published.

### Conflict of interest statement

The authors declare that the research was conducted in the absence of any commercial or financial relationships that could be construed as a potential conflict of interest.

## References

[B1] Afchain A. L. Carlin F. Nguyen-The C. Albert I. (2008). Improving quantitative exposure assessment by considering genetic diversity of *Bacillus cereus* in cooked, pasteurized and chilled foods. Int. J. Food Microbiol. 128, 165–173. 10.1016/j.ijfoodmicro.2008.07.028 18805600

[B2] Aklilu E. Tukimin E. B. Daud N. B. A. Kyaw T. (2016). Enterotoxigenic*Bacillus cereus* from cooked chicken meat: a potential public health hazard. Malays. J. Microbiol. 12, 112–115. 10.21161/mjm.70215

[B3] Aksu H. Bostan K. Ergün O. (2000). Presence of *Bacillus cereus* in packaged some spices and herbs sold in Istanbul. Pak. J. Biol. Sci. 3, 710–712. 10.3923/pjbs.2000.710.712

[B4] Altayar M. Sutherland A. D. (2006). *Bacillus cereus* is common in the environment but emetic toxin producing isolates are rare. J. Appl. Microbiol. 100, 7–14. 10.1111/j.1365-2672.2005.02764.x 16405680

[B5] Ågren J. Schäfer M. O. Forsgren E. (2017). Using whole genome sequencing to study American foulbrood epidemiology in honeybees. PLoS ONE 12:e0187924. 10.1371/journal.pone.0187924 29140998 PMC5687730

[B6] Andersson A. Ronener U. Granum P. E. (1995). What problems does the food industry have with the spore-forming pathogens *Bacillus cereus* and *Clostridium perfringens*? Int. J. Food Microbiol. 28, 145–155. 10.1016/0168-1605(95)00053-4 8750663

[B7] Ankolekar C. Rahmati T. Labbé R. G. (2009). Detection of toxigenic *Bacillus cereus* and *Bacillus thuringiensis* spores in U.S. rice. Int. J. Food Microbiol. 128, 460–466. 10.1016/j.ijfoodmicro.2008.10.006 19027973

[B8] Stenfors Arnesen L. P. Fagerlund A. Granum P. E. (2008). From soil to gut: *Bacillus cereus* and its food poisoning toxins. FEMS Microbiol. 32, 579–606. 10.1111/j.1574-6976.2008.00112.x 18422617

[B9] Bedi S. K. Sharma C. S. Gill J. P. S. Aulakh R. S. Sharma J. K. (2005). Incidence of enterotoxigenic *B. cereus* in milk and milk products. J. Food Sci. Tech. 42, 272–275.

[B10] Borch E. Arinder P. (2002). Bacteriological safety issues in red meat and ready-to-eat meat products, as well as control measures. Meat Sci. 62, 381–390. 10.1016/S0309-1740(02)00125-0 22061614

[B11] Carlin F. Brillani J. Brousso V. Clavel T. Duport C. Jobin M. (2010). Adaptation of *Bacillus cereus*, an ubiquitous worldwide-distributed foodborne pathogen, to a changing environment. Food Res. Int. 43, 1885–1894. 10.1016/j.foodres.2009.10.024

[B12] Claus D. Berkeley R. C. W. (1986). Genus *Bacillus*, in Bergey's Manual of Systematic Bacteriology, Vol 2, eds Sneath P. H. A. Mair N. S. Sharpe M. E. Holt J. G. (Baltimore, MD: The Williams and Wilkins), 1104–1139.

[B13] Das S. Surendran P. K. Thampuran N. (2009). PCR-based detection of enterotoxigenic isolates of *Bacillus cereus* from tropical seafood. Indian J. Med. Res. 129, 316–320. 19491426

[B14] Drobniewski F. A. (1993). *Bacillus cereus* and related species. Clin. Microbiol. Rev. 6, 324–338. 10.1128/CMR.6.4.324 8269390 PMC358292

[B15] Eglezos S. Huang B. Dykes G. A. Fegan N. (2010). The prevalence and concentration of *Bacillus cereus* in retail food products in Brisbane, Australia. Foodborne Pathog. Dis. 7, 867–870. 10.1089/fpd.2009.0469 20230270

[B16] Ehling-Schulz M. Messelhäusser U. (2013). Bacillus “next generation” diagnostics: moving from detection toward subtyping and risk-related strain profiling. Front. Microbiol. 4:32. 10.3389/fmicb.2013.00032 23440299 PMC3579190

[B17] Faille C. Bénézech T. Midelet-Bourdin G. Lequette Y. Clarisse M. Ronse G.. (2014). Sporulation of *Bacillus* spp. within biofilms: a potential source of contamination in food processing environments. Food Microbiol. 40, 64–74. 10.1016/j.fm.2013.12.004 24549199

[B18] Fendri I. Ben Hassena A. Grosset N. Barkallah M. Khannous L. Chuat V.. (2013). Genetic diversity of food-isolated *Salmonella* Strains through Pulsed Field Gel Electrophoresis (PFGE) and Enterobacterial Repetitive Intergenic Consensus (ERIC-PCR). PLoS ONE 8:e81315. 10.1371/journal.pone.0081315 24312546 PMC3849149

[B19] Floristean V. Cretu C. Carp Carare M. (2007). Bacteriological characteristics of *Bacillus cereus* isolates from poultry. Bull. USAMV-CN 64, 425–430. 10.15835/buasvmcn-vm:64:1-2:2458

[B20] Food Standards Australia New Zealand [FSANZ] (2001). Guidelines for Microbiological Examination of Ready-to-Eat Foods. Available online at: https://www.foodstandards.gov.au/code/microbiollimits/documents/Guidelines%20for%20Micro%20exam.pdf

[B21] Gilbert R. J. Kramer J. M. (1986). Bacillus Cereus Food Poisoning, in Progress, in Food Safety: Proceedings of Symposium, eds Cliver D. C. Cochrane B. A. (Madison, WI: Food Research Institute, University of Wisconsin-Madison), 85–93.

[B22] Granum P. E. (1994). *Bacillus cereus* and its toxins. Soc. Appl. Bacteriol. Symp. Ser. 23, 61S−66S. 8047911

[B23] Guinebretière M. H. Auger S. Galleron N. Contzen M. De Sarrau B. De Buyser M. L.. (2013). *Bacillus Cytotoxicus* sp. nov. is a novel thermotolerant species of the *Bacillus cereus* group occasionally associated with food poisoning. Int. J. Syst. Evol. Microbiol. 63, 31–40. 10.1099/ijs.0.030627-0 22328607

[B24] Guinebretière M. H. Thompson F. L. Sorokin A. Normand P. Dawyndt P. Ehling-Schulz M.. (2008). Ecological diversification in the *Bacillus cereus* Group. Environ. Microbiol. 10, 851–865. 10.1111/j.1462-2920.2007.01495.x 18036180

[B25] Guinebretière M. H. Velge P. Couvert O. Carlin F. Debuyser M. L. Nguyen-The C. (2010). Ability of *Bacillus cereus* Group to cause food poisoning varies according to phylogenetic affiliation (groups I to VII) rather than species affiliation. Environ. Microbiol. 10, 851–865. 10.1128/JCM.00921-10 PMC2937725 20660215

[B26] Havelaar A. H. Haagsma J. A. Mangen M. J. Kemmeren J. M. Verhoef L. P. Vijgen S. M.. (2012). Disease burden of foodborne pathogens in the Netherlands, 2009. Int. J. Food Microbiol. 156, 231–238. 10.1016/j.ijfoodmicro.2012.03.029 22541392

[B27] Harbottle H. White D. G. McDermott P. F. Walker R. D. Zhao S. (2006). Comparison of multilocus sequence typing, pulsed-field gel electrophoresis, and antimicrobial susceptibility typing for characterization of *Salmonella enterica* serotype Newport isolates. J. Clin. Microbiol. 44, 2449–2457. 10.1128/JCM.00019-06 16825363 PMC1489510

[B28] Hendriksen N. B. Hansen B. M. (2011). Diagnostic properties of three conventional selective plating media for selection of *Bacillus cereus, B. thuringiensis* and *B. weihenstephanensis*. Folia Microbiol. 56, 535–539. 10.1007/s12223-011-0079-0 22083787

[B29] Hill K. K. Ticknor L. O. Okinaka R. T. Asay M. Blair H. Bliss K. A.. (2015). Fluorescent amplified fragment length polymorphism analysis of *Bacillus anthracis, Bacillus cereus*, and *Bacillus thuringiensis* isolates. Appl. Environ. Microbiol. 70, 1068–1080. 10.1128/AEM.70.2.1068-1080.2004 14766590 PMC348840

[B30] Jan S. Brunet N. Techer C. Le Marechal C. Kone A. Z. Grosset N.. (2011). Biodiversity of psychotrophic bacteria of the *Bacillus cereus* group collected on farm and in egg product industry. Food Microbiol. 28, 261–265. 10.1016/j.fm.2010.05.029 21315982

[B31] Jiménez G. Urdiain M. Cifuentes A. López-López A. Blanch A. R. Tamames J.. (2013). Description of *Bacillus toyonensis* sp. nov. a novel species of the *Bacillus cereus* group, and pairwise genome comparisons of the species of the group by means of ANI calculations. Syst. Appl. Microbiol. 36, 383–391. 10.1016/j.syapm.2013.04.008 23791203

[B32] Kidd T. J. Grimwood K. Ramsay K. A. Rainey P. B. Bell S. C. (2011). Comparison of three molecular techniques for typing *Pseudomonas aeruginosa* isolates in sputum samples from patients with cystic fibrosis. J. Clin. Microbiol. 49, 263–268. 10.1128/JCM.01421-10. 21084517 PMC3020435

[B33] Kim K. Seo J. Wheeler K. Park C. Kim D. Park S.. (2005). Rapid genotypic detection of *Bacllus anthracis* and the *Bacillus cereus* group by multiplex real time PCR melting curve analysis. Immunol. Med. Microbiol. 43, 301–310. 10.1016/j.femsim.2004.10.005 15681162

[B34] Kneifel W. Berger E. (1994). Microbiological criteria of random samples of spices and herbs retailed on the Austrian market. J. Food Protect. 57, 893–901. 10.4315/0362-028X-57.10.893 31121691

[B35] Kramer J. M. Gilbert R. J. (1989). *Bacillus cereus* and other *Bacillus* species, in Foodborne Bacterial Pathogens, ed Doyle M. P. (New York, NY: Marcel Dekkar Inc), 21–70.

[B36] Lee H. Y. Chai L. C. Tang S. Y. Jinap S. Ghazali M. Nakaguchi Y. (2009). Application of MPN-PCR in biosafety of *Bacillus cereus* s.I. for ready-to-eat cereals. Food Control 20, 1068–1071. 10.1016/j.foodcont.2009.01.009

[B37] Lücking G. Stoeckel M. Atamer Z. Hinrichs J. Ehling-Schulz M. (2013). Characterization of aerobic spore-forming bacteria associated with industrial dairy processing environments and product spoilage. Int. J. Food Microbiol. 166, 270–279. 10.1016/j.ijfoodmicro.2013.07.004 23973839

[B38] Merzougui S. Lkhider M. Grosset N. Gautier M. Cohen N. (2013a). Differenciation by molecular typing of *Bacillus cereus* isolates from food in Morocco: PFGE-Eric PCR. J. Food Public Health 3, 223–227. 10.5923/j.fph.20130304.06

[B39] Merzougui S. Lkhider M. Grosset N. Gautier M. Cohen N. (2013b). Prevalence, PFGE typing and antibiotic resistance of *Bacillus cereus* group isolated from food in Morocco. Foodborne Pathog. Dis. 11, 145–149. 10.1089/fpd.2013.1615 24206436

[B40] Noller A. C. McEllistrem M. C. Stine O. C. Morris J. G. Jr Boxrud D. J. Dixon B.. (2003). Multilocus sequence typing reveals a lack of diversity among *Escherichia coli* O157: H7 isolates that are distinct by pulsed-field gel electrophoresis. J. Clin. Microbiol. 41, 675–679. 10.1128/JCM.41.2.675-679.2003 12574266 PMC149678

[B41] Oranusi U. S. Braide W. Osigwe G. A. (2012). Investigation on the microbial profile of canned foods. J. Biol. Food Sci. Res. 1, 15–18. Available online at: https://pdfs.semanticscholar.org/1d22/c342b1ebfe140efeef9912c03dfe658f930d.pdf

[B42] Pfrunder S. Grossmann J. Hunziker P. Brunisholz R. Gekenidis M. T. Drissner D. (2016). *Bacillus cereus* group-type strain-specific diagnostic peptides. J. Proteome Res. 15, 3098–3107. 10.1021/acs.jproteome.6b00216 27432653

[B43] Public Health England [PHE] (2009). Guidelines for Assessing the Microbiological Safety of Ready-to-Eat Foods Placed on the Market. Available online at: www.gov.uk/government/publications/ready-to-eat-foods-microbiological-safety-assessment-guidelines

[B44] Rahmati T. Labbe R. (2008). Levels and toxigenicity of *Bacillus cereus* and *Clostridium perfringens* from retail seafood. J. Food Prot. 71, 1178–1185. 10.4315/0362-028X-71.6.1178 18592743

[B45] Revazishvili T. Kotetishvili M. Stine O. C. Kreger A. S. Morris J. G. Jr. Sulakvelidze A. (2004). Comparative analysis of multilocus sequence typing and pulsed-field gel electrophoresis for characterizing *Listeria monocytogenes* strains isolated from environmental and clinical sources. J. Clin. Microbiol. 42, 276–285. 10.1128/JCM.42.1.276-285.2004 14715765 PMC321703

[B46] Rosenquist H. Smidt L. Andersen S. R. Jensen G. B. Wilcks A. (2005). Occurrence and significance of *Bacillus cereus* and *Bacillus thuringiensis* in ready-to-eat food. FEMS Microbiol. 250, 129–136. 10.1016/j.femsle.2005.06.054 16043311

[B47] Samapundo S. Heyndrickx M. Xhaferi R. Devlieghere F. (2011). Incidence, diversity and toxin gene characteristics of *Bacillus cereus* group strains isolated from food products marketed in Belgium. Int. J. Food Microbiol. 150, 34–41. 10.1016/j.ijfoodmicro.2011.07.013 21840614

[B48] Shah R. C. Wadher B. J. Bhoosreddy G. L. (1996). Incidence and characteristics of *Bacillus cereus* isolated from Indian foods. J. Food Sci. Tech. 33, 249–250.

[B49] Six S. C. Buyser M. Vignaud M. Dao T. T. Messio S. Payraud S. (2012). Toxi-infections alimentaires collectives à *Bacillus cereus* : bilan de la caractérisation des souches de 2006 à 2010. Bull. Épidémiol. Anim. Aliment. 50, 57–61. Available online at: http://invs.santepubliquefrance.fr//pmb/invs/(id)/PMB_10678

[B50] Sudershan R. V. Naveen Kumar R. Kashinath L. Bhaskar V. Polasa K. (2012). Microbiological hazard identification and exposure assessment of poultry products sold in various localities of Hyderabad, India. Sci. World J. 2012:736040. 10.1100/2012/736040 22593705 PMC3347783

[B51] Tewari A. Abdullah S. (2015). *Bacillus cereus* food poisoning: international and Indian perspective. J. Food Sci. Technol. 52, 2500–2511. 10.1007/s13197-014-1344-4 25892750 PMC4397285

[B52] Väisänen O. M. Mentu J. Salkinoja-Salonen M. S. (1991). Bacteria in food packaging paper and board. J. Appl. Microbiol. 71, 130–133. 1917722 10.1111/j.1365-2672.1991.tb02967.x

[B53] Valjevac S. Hilaire V. Lisanti O. Ramisse F. Hernandez E. Cavallo J. D.. (2005). Comparison of minisatellite polymorphisms in the *Bacillus cereus* complex: a simple assay for large-scale screening and identification of strains most closely related to *Bacillus anthracis*. Appl. Environ. Microbiol. 71, 6613–6623. 10.1128/AEM.71.11.6613-6623.2005 16269689 PMC1287610

[B54] Van Cauteren D. Le Strat Y. Sommen C. Bruyand M. Tourdjman M. Da Silva N. J.. (2017). Estimated Annual Numbers of Foodborne pathogen-associated illnesses, hospitalizations, and deaths, France, 2008-2013. Emerg. Infect. Dis. 23, 1486–1492. 10.3201/eid2309.170081 28820137 PMC5572882

[B55] Versalovic J. Koeuth T. Lupskil J. R. (1991). Distribution of repetitive DNA sequences in eubacteria and application to fingerprinting of bacterial genomes. Nucleic Acids Res. 19, 6823–6831. 10.1093/nar/19.24.6823 1762913 PMC329316

[B56] Walsh P. S. Metzger D. A. Higuchi R. (1991). Chelex 100 as a medium for simple extraction of DNA for PCR-based typing from forensic material. BioTechniques 10, 506–513. 1867860

[B57] Wijnands L. M. Dufrenne J. B. Rombouts F. M. in 'T. Veld P. H. Van Leusden F. M. (2006). Prevalence of potentially pathogenic *Bacillus cereus* in food commodities. in the Netherlands. J. Food Prot. 69, 2587–2594. 10.4315/0362-028X-69.11.2587 17133800

